# Drug Resistance in Cancers: A Free Pass for Bullying

**DOI:** 10.3390/cells11213383

**Published:** 2022-10-26

**Authors:** Jing Li, Xiao Li, Qie Guo

**Affiliations:** The Department of Clinical Pharmacy, The Affiliated Hospital of Qingdao University, Qingdao 266003, China

**Keywords:** drug resistance, tumor microenvironment, exosomes, metabolic reprogramming, glycosylation, autophagy

## Abstract

The cancer burden continues to grow globally, and drug resistance remains a substantial challenge in cancer therapy. It is well established that cancerous cells with clonal dysplasia generate the same carcinogenic lesions. Tumor cells pass on genetic templates to subsequent generations in evolutionary terms and exhibit drug resistance simply by accumulating genetic alterations. However, recent evidence has implied that tumor cells accumulate genetic alterations by progressively adapting. As a result, intratumor heterogeneity (ITH) is generated due to genetically distinct subclonal populations of cells coexisting. The genetic adaptive mechanisms of action of ITH include activating “cellular plasticity”, through which tumor cells create a tumor-supportive microenvironment in which they can proliferate and cause increased damage. These highly plastic cells are located in the tumor microenvironment (TME) and undergo extreme changes to resist therapeutic drugs. Accordingly, the underlying mechanisms involved in drug resistance have been re-evaluated. Herein, we will reveal new themes emerging from initial studies of drug resistance and outline the findings regarding drug resistance from the perspective of the TME; the themes include exosomes, metabolic reprogramming, protein glycosylation and autophagy, and the relates studies aim to provide new targets and strategies for reversing drug resistance in cancers.

## 1. Introduction

Cancer is one of the most serious diseases and the second leading cause of death globally [1]. The use of chemotherapeutic drugs, immune checkpoint inhibitors (ICIs), and molecular targeted inhibitors represents an optimal strategy for cancer therapy [2,3,4]. Unfortunately, drug resistance has become increasingly common and contributes to tumor metastasis and local recurrence [5]. Elucidating the underlying mechanisms that promote drug resistance and establishing effective strategies to overcome drug resistance have long been urgent issues in cancer treatment. A steady efflux of drugs is essential for addressing the aggressive biological behaviors of tumor cells that are resistant to chemotherapy [6]. Apoptosis resistance and the DNA repair response also contribute to the development of drug resistance [7,8]. It is recently documented that genetic variability in single tumor cells, also known as intratumor heterogeneity (ITH), can lead to “cellular plasticity”, which has received considerable attention [9,10]. The properties of cellular plasticity enable tumor cells to reversibly switch their phenotypes or states between drug resistance and drug sensitivity and then escape and survive therapeutic challenges [11]. More surprisingly, tumor cells with cellular plasticity create a favorable tumor microenvironment (TME) in which they acquire distinct resistance to therapeutic drugs by reinventing themselves with metabolic alterations and aberrant epigenetic modifications, thus evolving a strategy to avoid cell death [12,13]. Therefore, experts are becoming more willing to accept that these mechanisms, including drug efflux, apoptosis resistance, and DNA repair response, may comprise only one chapter in the story of drug resistance, as tumor cells are very good at embellishing themselves to adapt to ever-changing environments [14]. Here, we focus on determinants that promote drug resistance in the TME, including exosomes, metabolic reprogramming, protein glycosylation, and autophagy, considering their implications for the development of successful therapeutic strategies.

## 2. TME: A Breeding Ground for Drug Resistance

As reported previously, the bidirectional interactions between tumor cells and their surroundings form the TME. The TME consists of tangible components such as malignant cells, tumor-associated immune cells, cancer-associated fibroblasts (CAFs), cancer stem cells (CSCs), endothelial cells, and adipocytes [15]. Biochemical components, including cytokines and cell adhesion molecules (CAMs), are also present in the TME [16]. Herein, we provide a brief overview of cellular and molecular immunosuppressive networks in a tumor-supportive physical and chemical TME, especially the immune cellular and nonimmune cellular components of the TME, and their contribution to drug resistance.

### 2.1. Tumor-Associated Immune Cells in the TME

#### 2.1.1. Regulatory T Cells (Tregs) and Regulatory B Cells (Bregs)

It is now widely accepted that the Tregs and Bregs infiltrate tumors and suppress immune responses [17]. Tregs, an immunosuppressive subset of the CD4^+^ T-cell family, are characterized by the expression of the master transcription factor forkhead box protein p3 (Foxp3) [18]. CD4^+^CD25^+^Foxp3^+^ represents a classical combined marker of Tregs [19]. CD4^+^CD25^+^Foxp3^+^ Tregs are the preponderating T cells that respond to cancers, outpacing CD8^+^ cytotoxic T lymphocytes (CTLs) during drug resistance development [20]. In particular, Tregs dampened CTL translocation to the tumor cells through collaborations with M2 macrophages and CAFs [21]. Tregs also induced the escape of cancers from drug activity by promoting TGF-β release, which restrained the expression of cytolytic products from CTLs, including perforin, FasL, IFN-γ, and granzymes A and B [22].

Notably, aberrant expression of programmed cell death 1 (PD-1)/PD1 ligand 1 (PD-L1) and cytotoxic T lymphocyte antigen 4 (CTLA-4) has been identified to be involved in Treg induction [23]. TGF-β release caused by Treg induction promotes paralyzing infiltration of differentiated NK cells which acquire PD-1 receptors and exhibit protumor activities [24]. Currently, how Bregs cells facilitate drug resistance in cancers is controversial, although investigations have revealed similar functions of PD-1 and its ligands in Breg biology [25]. PD-L1^+^ Bregs have been identified within the IgA^+^ B cell population in mice bearing liver tumors, and anti-PD-L1 treatment induces hepatocellular carcinoma (HCC) regression [26]. PD-L1^+^ Bregs also mediate oxaliplatin resistance in prostate cancer, which can be overturned by PD-L1 blockade [27].

Immune checkpoint inhibitors (ICIs) targeting CTLA-4, PD-1, and PD-L1 have made a milestone significance in cancer immunotherapy [28]. However, resistance to ICIs has become common, and only a minority of patients achieve durable responses [29]. This has fueled a wave of research on the molecular mechanisms of tumor resistance to ICIs. It has been reported previously that TYRO3 expression was higher in the patients with lung cancer who were resistant to nivolumab, pembrolizumab, and atezolizumab than that in the patients whose tumor progression was controlled by anti-PD-1/PD-L1 therapy. Mechanistically, TYRO3 facilitated tumor resistance to those ICIs by supporting a “protumor TME” in which the M1/M2 macrophage ratio was reduced [30]. A pooled CRISPR screen was conducted in a B16 transplantable melanoma model. *Adar1*, which encodes adenosine deaminase, has been shown to impair PD-1 blockade by increasing tumor inflammation with concomitant impairment of IFN sensing [31]. Primary resistance to pembrolizumab in patients with metastatic melanoma has been confirmed to be associated with the defects in the IFN signaling pathway due to JAK1 and JAK2 truncating mutations [32]. JAK1/2 loss-of-function mutations are also found in patients with metastatic colon cancer who are resistant to PD-1 blockade [33]. Anti-CTLA-4-targeted inhibitors such as ipilimumab and tremelimumab, were tested for advanced gastro-esophageal cancer, but both of them showed limited success in phase Ⅱ clinical trials [34]. Ipilimumab and tremelimumab also demonstrated limited activity as single agents in lung cancer [35]. More efforts should be made to shed light on the molecular mechanisms of tumor resistance to ICIs targeting CTLA-4.

CD13 (aminopeptidase N) and CD73 were expressed in a large number of Tregs, which reduces the effectiveness of ICIs, including nivolumab, pembrolizumab, and tislelizumab. In particular, CD13^+^CD4^+^CD25^high^ Tregs exhibited stronger suppressive function than CD4^+^CD25^high^ Tregs that did not express CD13 and increased in abundance with tumor stage in non-small cell lung cancer (NSCLC) patients treated with pembrolizumab [36]. CD73^+^CD4^+^CD25^high^ Tregs exhibit immunosuppressive effects, which pave the way for melanoma cells to escape nivolumab, pembrolizumab, and tislelizumab treatment [37].

Certainly, tumor cells often exhibit immune tolerance, which is largely mediated by the activation of both Tregs and Bregs. However, more evidence should be generated regarding the drug resistance caused by Tregs and Bregs to develop novel ICIs for cancer therapy, although other putative immune checkpoints, such as LAG3, CD73, B7H3, TIM3, CD39, TIGIT, and adenosine A2A receptor, have attracted interest [38].

#### 2.1.2. Tumor-Associated Macrophages (TAMs)

TAMs can be derived from either CD34^+^ bone marrow progenitor cells, known as recruited macrophages, or the yolk sac, which are considered as resident macrophages [39]. As reported previously, milk-fat globule-epidermal growth factor-VIII (MFG-E8) from TAMs conferred CSC-amplified cisplatin (CDDP) resistance in breast cancer by manipulating the activation of the signal transduction and transcription activator 3 (STAT3) and sonic hedgehog signaling pathways [40]. TAMs also secrete the cytokine interleukin (IL)-10 and CC-chemokine ligand 2 (CCL2), thus enacting a three-pronged strategy to impact the activity of the C-Jun N-terminal kinases (C-JNK), STAT3, BCL-2 and PI3K/AKT/mTOR signaling pathways to mediate paclitaxel (PTX) and tamoxifen resistance in breast cancer [41,42]. Pancreatic ductal adenocarcinoma (PDAC) is often formed by well-polarized cells in which increased CD163^+^ TAM infiltration is identified. In particular, CD163^+^ TAMs secrete insulin-like growth factor 1 (IGF-1), which activates the Insulin/IGF-1 signaling pathway, thus resulting in gemcitabine resistance in PDAC [43]. Furthermore, the activation of PD-L1/PD-L2 expressing CD163^+^TAMs “resets” CD3^-^CD56^hi^CD16^-ve^ NK cell activation that mediates antibody-dependent cell-mediated cytotoxicity (ADCC) in Hodgkin lymphoma (CHL) and diffuse large B-cell Lymphoma (DLBCL) with doxorubicin (DOX)-based therapy [44]. TAMs also activate the STAT3 signaling pathway and inhibit the apoptosis of multiple myeloma (MM) cells through the JAK2 signaling pathway upon bortezomib treatment. In vivo cotreatment with an ATP-competitive JAK2 inhibitor significantly improved MM cells sensitivity to bortezomib [45]. It is becoming clear that drug-resistant subpopulations are more plastic in response to chemotherapeutic drugs, and TAMs even serve as decoys. Therefore, reducing the recruitment and loading of TAMs, attenuating the activity of downstream pathways in TAMs, and inhibiting the secretion of cytokines by TAMs may be workable strategies to reverse the chemoresistance of tumor cells.

#### 2.1.3. Myeloid-Derived Suppressor Cells (MDSCs)

MDSCs, a heterogeneous population of immature myeloid cells with immunosuppressive functions, are not present in the steady state in healthy individuals but appear in cancer [46]. The release of cytokines and growth factors in tumor cells, including IL-6, macrophage colony-stimulating factor (M-CSF), vascular endothelial growth factor (VEGF), and granulocyte colony-stimulating factor (GM-CSF), is responsible for the induction and activation of MDSCs within the TME [47]. MDSCs exert their immunosuppressive activities by inhibiting the production of immunosuppressive mediators, such as prostaglandin E2 (PGE2), arginase-1 (ARG1), and inducible nitric oxide synthase (iNOS), thus decreasing the intratumoral CD8^+^ T cell: Treg ratio, suppressing the cell cycle of T cells and restricting their recruitment into tumor sites [48]. In addition, MDSCs suppress CD8^+^ T-cell cytotoxic responses in an antigen-specific manner [49]. Recent studies have indicated that the abnormal activation of MDSCs contributes to the development of drug resistance in cancers. It has been suggested that 5-fluorouracil (5-FU) and gemcitabine promote inflammasome activation to induce the IL-1β production in MDSCs, which increases IL-17 secretion by CD4^+^ T cells to weaken anticancer efficacy of these chemotherapeutic drugs towards B16F10 melanoma, 4T1 mammary carcinoma, and Lewis lung carcinoma (LLC) [50]. Furthermore, MDSCs with a polymorphonuclear structure and neutrophils in bone marrow were found to promote DOX resistance in breast cancer through the secretion of IL-1β, which in turn encouraged PI3K/RAC and IL-1RI/β-catenin-dependent BIRC3 transcription [51]. Interestingly, GM-CSF encourages the survival of MDSCs via a pSTAT5-dependent pathway and protects them from the effects of sunitinib, thus promoting the resistance to sunitinib in metastatic renal cell carcinoma [52]. Additionally, CD11b^+^CD14^+^S100A9^+^ MDSCs suppress CD8^+^ T cells via iNOS, arginase, and the IL-13/IL-4Rα axis to promote CDDP resistance in NSCLC [53]. It has been proven that population alterations of L-arginase-and iNOS-expressed CD11b^+^CD14^-^CD15^+^CD33^+^ MDSCs and CD8^+^ T lymphocytes were associated with DOX and melphalan resistance in MM patients [54]. The level of MDSCs is also related to patient responses to CTLA-4/ipilimumab [55,56] and PD-1 inhibition [57,58].

#### 2.1.4. Tumor-Associated Dendritic Cells (TADCs)

Dendritic cells (DCs), capable of priming both naïve and memory T cells, play a critical role in initiating and regulating tumor-specific immune responses [59]. Unfortunately, TADCs are usually immature and dysfunctional in the TME, and harbor robust immunosuppressive potential by increasing Tregs and MDSCs [60] and suppressing the activation of cytotoxic CD8^+^ T cells, for example, by expressing indoleamine-2,3-dioxygenase (IDO1), ARG, and PD-L1 and secreting TGF-β and IL-10 [61]. TADCs were also postulated to be involved in chemoresistance. The recruitment of dysfunctional tumor-infiltrating PD-1^+^ TADCs with high expression of inflammatory cytokines/chemokines and PD-L1 weakens the immunotherapeutic response by PD-1/PD-L1 blockade and induces pembrolizumab resistance in cervical cancer [62]. Increased TADC-derived CXCL1/GRO and S100A8/A9 were associated with DOX/cyclophosphamide resistance in breast cancer [63]. TADC-derived *RESISTIN* promoted epithelial mesenchymal transformation (EMT) in lung cancer, which is a well-known mechanism underlying chemoresistance development by activating the Wolf–Hirschhorn syndrome candidate 1/Twist pathway [64]. TADC-derived *AMPHIREGULIN* induces STAT3 and AKT activation, which increases the expression of the EMT markers SNAIL and TWIST, indicating that the collaboration of STAT3 and AKT plays a crucial role in TADC-mediated chemoresistance in lung cancer [65].

### 2.2. Nonimmune Cells in the TME

#### 2.2.1. CAFs

CAFs are the most abundant stromal cells within the TME. It is well documented that CAFs exhibit exuberant proliferative ability and abundant secretion of inflammatory ligands, growth factors, and extracellular matrix (ECM) proteins, including cyclooxygenase-2 (COX2), PGE2, hepatocyte growth factor (HGF), VEGF, and α-smooth muscle actin (α-SMA) [66,67]. A groundbreaking report confirmed that high expression of CAF-specific VEGFA, PGE2S, COX2, EGFR, CCL2, and NANOG was considered as the culprit of CDDP resistance development in head and neck squamous cell carcinoma (HNSCC) [68]. Growing evidence indicates that the number of CAFs in chemoresistant gastric cancer (GC) patients is distinctly higher than that in chemosensitive GC patients. The enrichment of CAFs in chemoresistant GC patients was verified using α-SMA. α-SMA^+^ CAFs secrete IL-11 to activate the JAK/STAT3/BCL-2 signaling pathway, thereby potentiating CDDP, DOX, and etoposide resistance in GC [69]. Furthermore, CAF-derived IL-8 caused CDDP resistance in GC by promoting NF-κB activation and P-glycoprotein (P-gp) upregulation, which increased the levels of PI3K, phospho-AKT (p-AKT), and phospho-p65 (p-P65) [70]. Conditioned medium (CM) from CAFs induced the translocation of AKT, P38, and survivin to the nucleus, thus promoting oxaliplatin and 5-FU resistance in colorectal cancer (CRC) [71]. Another CRC study also reported a significant correlation between a large proportion of α-SMA-expressing CAFs and resistance to 5-FU plus oxaliplatin-based chemotherapy [72]. Paracrine-secreted plasminogen activator inhibitor-1 (PAI-1) from CDDP-treated CAFs activated the AKT and ERK1/2 signaling pathways and suppressed caspase-3-mediated apoptosis, thereby promoting CDDP resistance in esophageal squamous cell carcinoma [73].

Tyrosine kinase inhibitors (TKIs) such as lapatinib and gefitinib, were usually used to treat breast cancer. It has been identified that most of the non-cell-autonomous mechanisms of lapatinib and gefitinib resistance involve the action of CAFs. Interestingly, lapatinib resistance was influenced by breast ductal carcinoma cells being spatially adjacent to CAFs, since anti-apoptotic BCL-2/BCL-XL, PI3K/AKT, and JAK/STAT signaling pathways were induced in lapatinib-treated tumor cells. This induction was associated with CAF-induced hyaluronic-acid-stimulated stromal protection and JAK/STAT-mediated intercellular communication between tumor cells and CAFs [74]. Another interesting study suggested that the HGF/MET signaling pathway in CAFs was activated, and secreted HGF conferred gefitinib resistance in breast cancer by increasing MET phosphorylation [75]. Furthermore, as a result of CAF activation, the estrogen/GPER/cAMP/PKA/CREB signaling pathway is activated, which switches breast cancer cells to aerobic glycolysis and provides them with extra pyruvate and lactate to survive epirubicin treatment [76]. 

Finally, novel interventional strategies targeting TAMs have significant potential to improve the efficacy of chemotherapeutic and/or immunotherapeutic treatments.

#### 2.2.2. CSCs

CSCs with self-renewal and tumor-initiating abilities play a vital role in the development of drug resistance. Overexpression of ATP binding cassette (ABC) transporters, which mediate drug efflux and promote chemoresistance in breast cancer, has been regarded as a hallmark of CSCs. In particular, the protein level of P-gp has been positively associated with CD44 expression, which is a biomarker of CSC presence in breast cancer [77]. The self-renewal of liver cancer stem cells (LCSCs) was characterized by increased expression of toll-like receptor 4 (TLR4), which led to CDDP, 5-FU, and DOX resistance in HCC [78]. TLR4 is also expressed in glioma CD133^+^ CSCs and TLR4 interaction with lipopolysaccharide (LPS) confers adriamycin (ADR) resistance in glioma [79]. Similarly, CSCs express TLR2 and its ligand high-mobility group protein 1 (HMGB1), and this autocrine loop participates in DOX resistance development in breast cancer [80].

It has been shown that CSC-expressing ALDH is also involved in the development of chemoresistance. The expression of ALDH in CSCs is positively correlated with carboplatin resistance in ovarian cancer, in which ALDH1 activates the cell cycle checkpoint and the DNA repair network [81]. Similarly, high expression of ALDH1 is positively associated with the level of CSC-signaling-related genes, including SOX9, SHH, and HES-1, thus conferring 5-FU and DOX resistance in esophageal cancer [82]. Moreover, CD44^+^ CSCs highly express ALDH and exhibit distinct resistance to CDDP in pleural mesothelioma [83].

The inhibitor of differentiation/DNA-binding (ID), a member of the helix–loop–helix (HLH) transcription factor family, is mainly expressed in CSCs and regulates the transcription of genes related to cell differentiation [84]. Knockdown of the *ID1* gene restores the decreased self-renewal ability of CSCs, followed by the reversal of chemoresistance in GC, in which the destruction of the NANOG/octamer-binding protein 4 (OCT-4) signaling pathway is involved [85]. ID1 upregulation also abrogates differentiation signals in GSCs and contributes to temozolomide resistance in glioblastoma (GBM) [86]. Musashi is an RNA-binding protein that maintains the unlimited proliferation of CSCs by exerting posttranscriptional regulation of stress resistance genes [87]. Musashi-1 (MSI1) robustly promoted the secretion of IL-6 in an autocrine/paracrine manner, which was governed by AKT activity. MSI1 protected GBM cells from CDDP-induced apoptosis through the AKT/IL-6 regulatory circuit [88]. Additionally, MSI1 is an important factor in the generation of CD44^+^ CSCs and enhances 5-FU resistance in CRC by inducing the formation of anti-apoptotic stress granules (SGs) [89]. Musashi-2 (MSI2) upregulates the expression of the “self-renewal gene” Lin28A in LCSCs, thereby increasing the levels of the pluripotency factors NANOG, OCT-4, and SOX2 to promote sorafenib resistance in HCC [90]. Dual knockdown of the *MSI1* and *MSI2* genes attenuates CSC characteristics, accompanied by reduced NOTCH, MYC, and ALDH4A1 levels, and eventually reverses PTX resistance in ovarian cancer [91].

#### 2.2.3. Endothelial Cells

High levels of tumor endothelial marker-7 (TEM-7), vascular endothelial-cadherin and CD31 encouraged NF-κB-dependent AKT activation and VEGF expression in tumor-associated endothelial cells (TAECs), thereby inducing gemcitabine resistance in HCC [92]. Forced expression of survivin in TAECs protects these cells from chemotherapy-induced apoptosis mediated by caspase-4/7 activation, thus promoting etoposide, PTX, and temozolomide resistance in malignant glioma [93]. The transformation of TAECs into mesenchymal stem cell (MSC)-like cells drives the c-MET-mediated axis that activates the β-catenin/WNT signaling pathway and induces multidrug-resistance-associated protein 1 (MRP1) expression and temozolomide resistance in GBM [94].

#### 2.2.4. Adipocytes

Emerging evidence has suggested that cancer-associated adipocytes (CAAs) provide fuels for cancer cells in the form of free fatty acids and amino acids, thus mediating apoptosis resistance and chemoresistance in acute lymphoblastic leukemia (ALL). CAAs protected ALL cells from vincristine-induced apoptosis by upregulating the expression of BCL-2 and PIM-2 (a pro-survival oncogene), thus promoting vincristine resistance [95]. High-dose glucocorticoids (GCs) are usually used in the treatment of aggressive forms of chronic lymphocytic leukemia (CLL). Fatty acid oxidation (FAO) in CAAs causes CLL cells to be resistant to dexamethasone by inhibiting PPAR-mediated apoptosis [96]. CAAs parameters also profile the TME and promote the growth and local infiltration of potential lesions in breast cancer [97].

### 2.3. Cytokines in the TME

CAFs and TAMs are the major cell types in the TME that secrete IL-6 and activate STAT3 in cancers [98]. CAFs promote paclitaxel resistance by encouraging EMT progress through the IL-6/JAK2/STAT3 pathway [99]. Maladjusted miR-155-5p/C/EBPβ/IL-6 signaling in TAMs enhanced 5-FU and oxaliplatin resistance in CRC cells by regulating the IL-6R/STAT3/miR-204-5p axis and increasing the expression of antiapoptotic proteins [100].

IL-6 is one of the most important survival factors in MM. Serum IL-6 induces HO-1 upregulation by activating the JAK2/STAT3 signaling pathway and promotes the overexpression of the antiapoptotic gene BCL-XL, thus causing lenalidomide resistance in MM cells [101]. IL-6 upregulates the expression of multidrug resistance gene 1 (MDR-1) and glutathione transferase (GST-π), thus facilitating drug efflux which is regarded as an important cause of multidrug resistance (MDR) development in ovarian cancer. IL-6 also activates the EGFR/AKT/NF-κB and EGFR/MEK/NF-κB signaling pathways to induce the expression of apoptosis suppressor proteins, including BCL-2, BCL-XL, and X-linked apoptosis suppressor protein (XIAP), thus inducing resistance to tocilizumab in combination with platinum in ovarian cancer cells [102].

TAEC-derived VEGF induces DOX resistance in soft tissue sarcoma without overtly affecting tumor cells, but promotes the proliferation and migration of TAEC, indicating that TAECs facilitate DOX resistance by promoting vascular abnormalities [103]. VEGF overexpression also inhibited cell apoptosis by interacting with the activation of the STAT1 and STAT3 signaling pathways to upregulate the expression of myeloid leukemia genes 1(MCL-1) and XIAP, thereby conferring resistance to chemotherapeutic drugs in B lymphoblastoma [104].

NF-κB-dependent HGF production by CAFs activates Met-dependent signaling. Lactic acid levels in the environment promoted HGF production by CAFs, as well as acquired resistance to EGFR-TKIs in NSCLC [105]. In particular, CAF-derived HGF mediates intrinsic resistance to afatinib in lung cancer by activating the MET/PI3K/AKT and MET/MAPK/ERK signaling pathways and triggering the progression of EMT and, chemoresistance development [106]. HGF also encourages the phosphorylation of c-MET, thus facilitating the generation of gefitinib resistance in lung cancer [107].

### 2.4. CAMs in the TME

CAMs refer to a family of transmembrane glycoproteins that act as messengers connecting the exterior and interior of cells, among which integrins are best qualified to promote the drug resistance of tumor cells [108]. A decidedly different response to chemotherapeutic drugs was identified between breast cancer cells cultured under ordinary conditions and breast cancer cells cultured in a three-dimensional matrix, suggesting that tumor cells responded differently to therapeutic drugs depending on the TME. More interestingly, blocking the ubiquitous recognition of integrin β1 rendered HER2^+^ breast cancer cells sensitive to trastuzumab [109]. The overexpression of integrinα5β1 guides temozolomide resistance in glioma by interfering with the p53 signaling pathway [110].

### 2.5. The Hypoxic TME

TAMs show more M2-like phenotypes in hypoxic TME. M2 phenotype TAMs respond to IL-6-receptor-mediated signals, particularly tyrosine phosphorylation of STAT3, and are responsible for the pro-survival adaptation of tumor cells to hypoxia [111]. IL-6/STAT3 activation in the TAMs also enhances the expression of Rab family proteins to facilitate CDDP resistance in ovarian cancer [112].

Tumor cells treated with anticancer drugs in a hypoxic environment are also prone to developing resistance to cancer therapy. Cycling hypoxia also induced ROS-mediated activation of hypoxia-inducible factor 1alpha (HIF-1α) and NF-κB, which was associated with increased expression of the antiapoptotic protein BCL-XL in GBM cell lines and xenograft tumors, resulting in temozolomide resistance in GBM [113]. Most hypoxic cells undergo cell cycle arrest in G1/S phase through HIF-1α-dependent regulation of P27, while most anticancer drugs kill tumor cells with rapid proliferation [114]. The dizzying pace of hypoxic p27 induction significantly activates DNA repair enzymes that repair the DNA damage caused by platinum and alkylating drugs [115]. HIF also causes the upregulation of P-gp, multidrug-resistance-associated proteins (MRPs), lung resistance protein (LRP), and ABC subfamily G member 2 (ABCG2), which increase drug efflux and reduce intracellular drug concentrations, thereby facilitating sorafenib resistance in HCC [116]. Additionally, hypoxia is associated with a higher risk of p53 gene mutation, which activates the JAK2/STAT3 signaling pathway, thus leading to gemcitabine resistance in pancreatic cancer [117].

**Figure 1:**
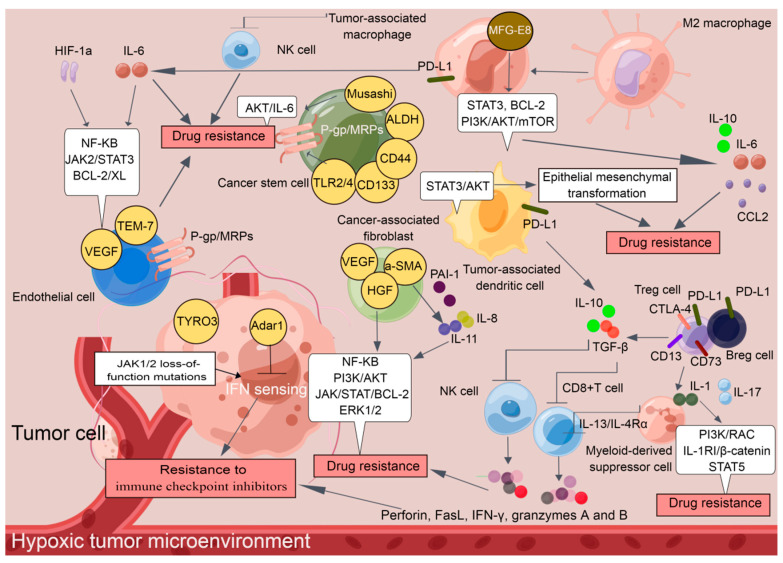
A tumor-supportive TME facilitating the development of drug resistance in tumor cells (*By Figdraw*).

These findings demonstrate that immunosuppressive cells and nonimmune cells seem to have their own functions and actually cooperate with each other to establish a tumor-supportive TME, which harbors tumor cells and facilitates the development of drug resistance (Figure 1). The cellular and molecular components of the TME play critical roles in determining the response rate and clinical outcomes to cancer therapy. Although additional effort is required before TME-targeting molecules can be applied clinically, an understanding of non-cell-autonomous resistance driven by TME can lead to novel combinations of currently available antitumor agents.

## 3. Exosomes: A Critical Factor in Drug Resistance Development

Exosomes are small (30–150 nm) extracellular vesicles (EVs) containing regulatory RNAs, such as microRNAs (miRNAs), circular RNAs (circRNAs), long non-coding RNAs (lncRNAs), and protein cargo; they are constantly secreted by all cells and involved in the intercellular transportation of materials [118]. To date, numerous studies have confirmed that exosomes can trigger drug resistance.

### 3.1. Exosome-Mediated Drug Efflux

As early as 2003, it was identified that EVs which were newly recognized as exosomes expel DOX into the extracellular medium, thereby reducing the intracellular concentration of drugs. Consistently, the expression of genes related to EV shedding was positively correlated with DOX resistance in breast cancer [119]. The increased number of EVs in CAAs also contributed to the ADR resistance in breast cancer [120].

Recently, tumor-derived exosomes (TDEs), which are involved in the process of drug resistance, have attracted much attention. The associated phenotypic changes and the potential for resistance transfer via TDEs have been evaluated, and the results indicate that intercellular transfer of P-gp enables drug-sensitive cells to develop DOX resistance in breast cancer [121]. P-gp transfer via TDEs gradually increases with longer coculturing of drug-resistant and sensitive strains, thus mediating DOX resistance in bladder cancer [122]. Dysregulation of Rab8B and Rab5 accelerates exosome-mediated P-gp intercellular transfer to advance vincristine resistance in oral epidermoid carcinoma [123]. Moreover, the interaction between miR-1246 and caveolin-1 (CAV1) via TDEs caused PTX resistance in ovarian cancer by upregulating the expression of *ABCB1*, which is the gene encoding P-gp [124]. TDE-derived lncRNA XIST also upregulates *ABCB1* expression by binding to miR-124, thus mediating MDR in CRC [125]. Additionally, lncRNA VLDLR within TDEs upregulates ABCG2 expression, thus inducing sorafenib, camptothecin, and DOX resistance in HCC [126]. The overexpressed TDE-derived circNFIX promotes temozolomide resistance in glioma by increasing ABCG2 expression through the circNFIX/miR-132 axis [127].

Collectively, these findings demonstrate that exosome-mediated drug efflux is an ideal means by which cancerous cells acquire drug resistance.

### 3.2. Exosome-Mediated Apoptosis Resistance

Drug-resistant tumor cells are often accompanied by the downregulation of apoptotic proteins or the upregulation of anti-apoptotic proteins. Exosomes secreted by drug-resistant cells can transmit drug resistance to adjacent cells. TDEs also facilitated apoptosis resistance in drug-resistant tumor cells. Exosome-secreted lncRNA AFAP1-AS1 in trastuzumab-resistant cells that exhibited apoptosis resistance was significantly increased compared with that in drug-sensitive cells. AFAP1-AS1 upregulation caused by H3K27ac modification enhances the translation of ERBB2 by binding to a mRNA decay factor AU-rich binding factor 1 (AUF1), which leads to trastuzumab resistance in breast cancer [128]. Exosome-secreted miR-22 from CD63^+^ CAFs downregulates estrogen receptor-α (ERα) and PTEN expression, which leads to nuclear AKT activation, suppression of cell apoptosis, and eventually tamoxifen resistance in breast cancer [129]. Similarly, a high level of miR-21was found in exosomes secreted by CAAs and CAFs in advanced ovarian cancer. Interestingly, miR-21 is dynamically distributed in CAAs, CAFs, and tumor cells. Overloading of miR-21 inhibits the transcription of apoptotic protease activating factor-1 (APAF-1), which is considered to be a factor that promotes caspase-3/9-mediated apoptosis. These findings suggest that exosome-derived miR-21 promotes PTX resistance in ovarian cancer [130].

It can be inferred that exosome-mediated apoptosis resistance also contributes to drug resistance, in which transcriptional regulation targeting pro-apoptotic proteins via lncRNAs and miRNAs is involved. 

### 3.3. Exosome-Mediated DNA Repair

Chemotherapy induces DNA damage, but DNA repair contributes to drug resistance. Exosome-secreted lncRNA SBF2-AS1 upregulates the expression of X-ray repair cross-complementing protein 4 (XRCC4) through miR-151a-3p targeting, which accelerates double-strand break (DSB) repair and promotes the drug resistance of GBM cells to temozolomide [131]. Exosome-secreted miR-21-3p, miR-21-5p, and miR-891-5p increased the expression of XRCC1, which mediated DNA repair and carboplatin resistance in ovarian cancer [132]. Exosome-mediated DNA repair also promotes the survival of tumor cells encountering genotoxic stress. TDEs from breast cancer cells receiving radiotherapy triggered the DNA damage repair response (DDR) such as ataxia telangiectasia-mutation (ATM) by increasing the phosphorylation of histone H2AX and checkpoint kinase 1 (CHK1) in recipient cells [133]. Interestingly, TDEs promote the resistance of adjacent cells to radiation in HNSCC by inducing DSB repair, and radiation in turn increases the cellular uptake of exosomes through CD29/CD81 complex formation [134]. Taken together, exosome-mediated DNA repair cannot be ignored when investigating the underlying mechanisms involved in the development of drug resistance.

### 3.4. Exosome-Mediated Immune Escape

Immune escape is a major obstacle for cancer therapy, and exosomes are heavily characterized. TDE-mediated neutralization of molecular targeted inhibitors is a novel mechanism that should be investigated further. Exosome-derived CD20 modulated by the lysosome-related organelle-associated ATP-binding cassette (ABC) transporter A3 (ABCA3) has been proven to directly bind to rituximab, resulting in a reduction in cellular drug concentrations and rituximab resistance in CLL [135]. Exosome-derived EGFR also directly binds to cetuximab and suppresses its activity, which is one of the underlying mechanisms involved in the resistance of oral squamous cell carcinoma to cetuximab [136].

NK cells exert ADCC, by which monoclonal antibodies exert their therapeutic effects. TGF-β1 derived from murine mammary tumor exosomes acts as a “cell cycle retardant” by suppressing the expression of JAK3 and cyclin D3 in NK cells. Additionally, TGF-β1 overloading evidently reduces the release of perforin, thereby inhibiting the cytolytic activity of NK cells [137]. Alternatively, a hypoxic TME in lung cancer is usually established. Exosomes derived from these hypoxic tumor cells carry immunosuppressive molecules, especially TGF-β1 and miR-23a, which impair the cytotoxicity of NK cells and trigger bevacizumab resistance in lung cancer [138].

Complement has been shown to enhance the antitumor activity of molecular-targeted inhibitors by encouraging the formation of membrane attack complexes (MACs), which are strong drivers of cell lysis. TDEs contain extensive kinase casein kinase 2 (CK2), which phosphorylates complement C9 and protects B lymphoma cells from complement-dependent cytotoxicity (CDC), thereby inducing rituximab resistance [139]. Mortalin/GRP75 from oligodendroglioma-derived exosomes promotes the abscission of membrane vesicles loaded with MAC and protects tumor cells from temozolomide-mediated cytotoxicity [140].

Emerging evidence has revealed that TDEs also attenuate the cytotoxicity of adaptive immune cells. NKG2D expression is a hallmark of the activation of CD8^+^ and γδ^+^ T cells, and deficiency of the NKG2D receptor represents an important mechanism promoting immune escape [141]. TGF-β1-neutralizing Ab strongly abrogated NKG2D downmodulation, and TDE-derived TGF-β1 decreased the expression of NKG2D, which weakened the ability of CD8^+^ and γδ^+^ T cells to recognize tumor-associated antigens (TAAs) [142]. CAF-derived TGFβ1 also induces the miR-183 expression to inhibit the activation of DNAX activating protein 12 (DAP12) that represents a signal adaptor for lytic function in NK cells [143]. TDE-expressed NKG2D ligands, including the major histocompatibility complex class I polypeptide-related sequence A and B (MICA/B) and a disintegrin and metalloproteinase domain-containing protein 10 (ADAM10), are also considered as the “pseudoligands” to neutralize NKG2D in neuroblastoma [144]. CD8 ^+^ T cells in circulating tumor cells (CTCs) distinctly express FAS and PD-1. Unfortunately, TDEs carrying FasL and PD-L1 have a special preference for CD8^+^ T cells, which undergo apoptotic death, thus facilitating pembrolizumab resistance in NSCLC [145]. TDEs also promote the transformation of CD4^+^CD25^neg^ T cells into CD4^+^CD25^high^Foxp3^+^ Tregs by secreting FasL, IL-10, TGF-β1, CTLA-4, and granzyme B, thus promoting immune escape [146]. Moreover, exosome-derived miR-280b promotes the proliferation of Treg cells by directly targeting programmed cell death protein 4 (PDCD4), thereby causing oxaliplatin resistance in CRC [147]. Therefore, digging deeply into exosome-mediated immune escape will be helpful for elucidating the mechanisms of drug resistance development in cancers.

### 3.5. Exosome-Mediated EMT

Exosomes are regarded as widespread mediators of EMT. Exosome-derived “pro-EMT” factors are considered to be important forces triggering drug resistance [148]. Exosome-derived miR-92a-3p from CAFs encourages EMT by targeting F-box with 7 tandem WD40 (FBXW7) and modulator of apoptosis 1 (MOAP1), which further promotes the resistance to fluorouracil and oxaliplatin of colorectal cancer cells (CRCs) [149]. Alternatively, exosomal miR-500a-3p promotes CDDP resistance by negatively regulating FBXW7 in GC [150]. Exosome-derived miR-9-5p, miR-195-5p, and miR-203a-3p from CAFs and TDEs targeted the transcription factor one cut homeobox 2 (ONECUT2), promoting an increase in stemness and the expression of NOTCH1, SOX9, NANOG, OCT4, and SOX2. E-cadherin downregulation and the upregulation of N-cadherin and vimentin characterized EMT initiation in CSCs with NOTCH1, SOX9, NANOG, OCT4, and SOX2 overexpression, thus promoting DOX and PTX resistance in breast cancer [151].

**Figure 2:**
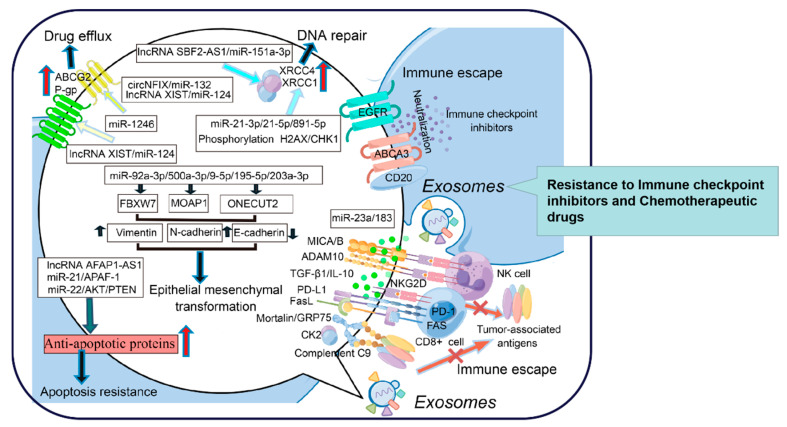
The role of exosomes in promoting drug resistance in tumor cells (*By Figdraw*).

TDEs can mediate the direct efflux of drugs, transfer drug transporters between tumor cells, regulate the expression of MDR genes, and become the bait target of therapeutic antibodies to reduce the concentration of drugs within and between cells. TDEs also transmit anti-apoptotic signals between tumor cells, promote DNA repair, and increase the survival rate of tumor cells after exposure to chemotherapy drugs or radiation, which destroy DNA. In addition, exosomes weaken the function of the immune system and promote tumor progression. Exosome-mediated EMT and CSC-like phenotypes are involved in the process of tumor progression and drug resistance. However, drug resistance is not caused by one or a few mechanisms; it is the result of the joint action of internal and external factors. Herein, exosome studies will provide some clues for elucidating the mechanisms of drug resistance (Figure 2). Collectively, exosomes have become attractive research objects due to their intriguing functions in intercellular communication and cell signaling. Advancements have been made in terms of understanding the biological functions of these EVs and using them for practical applications, such as the development of advanced therapeutics for reversing drug resistance.

## 4. Metabolic Reprogramming: The Genesis of Drug Resistance

Metabolic reprogramming is one of the hallmarks of tumorigenesis and has great relevance for therapeutic resistance in cancers [152]. Overstimulation of glycolysis, enhanced mitochondrial biosynthesis and fatty acid metabolism, and activation of the pentose phosphate pathway are regarded as important factors accelerating drug resistance development.

### 4.1. Overstimulation of Glycolysis

The energy produced by glycolysis is much less than that produced by oxidative phosphorylation (OXPHOS), but glycolysis, which can quickly provide ATP, supports tumor cells undergoing rapid proliferation. The preferential dependence of tumor cells on glycolysis is termed the Warburg effect [153]. Glycolysis is ideal for meeting the high demands of tumor cells for lactic acid, which promotes angiogenesis, metastasis, and immune escape, thus favoring malignant behaviors such as resistance to radiotherapy and chemotherapy [154,155,156]. Metabolomic and proteomic analyses showed that the levels of glycolytic intermediates, including glucose-6-phosphate, glyceraldehyde-3-phosphate, and phosphoenolpyruvate, in lapatinib-resistant BT-474-J4 cells were higher than those in parental BT-474 cells. These drug-resistant cells contained a large amount of lactic acid and pyruvate, indicating that lapatinib resistance in breast cancer was accompanied by phosphorylation-mediated reprogramming of glycolysis [157]. Lactate dehydrogenase-A (IDHA) catalyzes the transformation of pyruvate into lactic acid, thus promoting carcinogenesis [158]. IDHA depletion reduces the number of “Ras-generated CSCs”, suggesting that IDHA is a feasible therapeutic target for drug-resistant NSCLC [159]. Mitochondrial calcium uptake1 (MCU1/CBARA1), a gatekeeper of mitochondrial Ca^2+^ uptake, has been shown to drive aerobic glycolysis in ovarian cancer. MCU1 depletion increases oxygen consumption and reduces lactic acid production, which not only inhibits tumor growth, migration, and invasion but also increases the overall efficacy of CDDP in ovarian cancer patients [160]. Furthermore, the amount of lactic acid produced by the lymphoma cell lines Raji and HCT116 cultured under hypoxic conditions was significantly higher than that in cells cultured under normal conditions, indicating that glycolytic activity in tumor cells increased under hypoxia. Such metabolic adaptation was accompanied by ADR and cytarabine resistance in Burkitt lymphoma and B-cell lymphoma [161,162]. In addition, polypyrimidine tract-binding protein 1 (PTBP1) is a critical mediator of glycolysis. PTBP1 depletion can overcome the resistance of CRCs to vincristine and oxaliplatin [163]. Hexokinase 2 (HK2) is a rate-limiting enzyme that catalyzes glycolysis by promoting the immediate utilization of ATP. 2-Deoxy-D-glucose (2-DG), a well-known inhibitor of glycolysis, sensitizes tumor cells to ADR and PTX in human osteosarcoma and NSCLC by suppressing HK2 expression [164].

### 4.2. Enhancement of Fatty Acid Metabolism

#### 4.2.1. ATP Citrate Lyase (ACLY)

The metabolism of fatty acids in tumor cells is often ignored. However, it has been recognized that fatty acid synthesis in cancers is closely related to chemoresistance [165]. ATP citrate lyase (ACLY) is a rate-limiting enzyme catalyzing *de novo* fatty acid synthesis. It advances the transformation of citrate into oxaloacetic acid and acetyl coenzyme A, thus building a bridge connecting glucose metabolism and fatty acid metabolism. ACLY expression is upregulated in many cancers, including CRC, prostate cancer, bladder cancer, HCC, and GC. It has been reported that high expression of phosphorylated ACLY guides tumor staging and differentiation and predicts a poor prognosis in lung cancer [166]. Previously, the CRC cell line HT29 with overexpression of exogenous ACLY demonstrated significant resistance to SN38, which is known as an active metabolite of irinotecan. ACLY depletion with small interfering RNA or the small-molecule inhibitor GSK165 promotes HT29 chemosensitivity to SN38, indicating that ACLY amplifies irinotecan resistance in CRC [167]. Thus, ACLY is regarded as a central metabolic enzyme in cancer and may be a putative target for overcoming drug resistance.

#### 4.2.2. Fatty Acid Synthase (FASN)

FASN is another enzyme that participates in de novo fatty acid synthesis by guiding the condensation of the carbon skeleton into fatty acids. FASN overexpression is closely associated with acquired resistance to ADR and mitoxantrone in breast cancer and gemcitabine resistance in PDAC, respectively [168,169]. Orlistat, a FASN inhibitor, renders OCI-AML3 leukemia cells more sensitive to ABT-737 treatment when cultured alone or cocultured with the trophoblasts of bone-marrow-derived mesenchymal cells [170]. Recently, there has been increasing interest in the finding that apoptosis resistance induced by FASN contributes to chemoresistance development in cancers [171]. FASN overexpression protects prostate epithelial cell iPrECs from camptothecin-induced apoptosis [172]. FASN expression was negatively correlated with the number of apoptotic LNCaP-LN3 cells, which significantly increased after the administration of FAS inhibitors, including Fasnall, GSK2194069, and TVB-3166 [173]. FASN also induced the resistance of breast cancer cells to CDDP-induced apoptosis [174]. Furthermore, aberrant expression of FASN induces GC resistance to anoikis, which is termed a type of apoptosis, in response to inappropriate cell/ECM interactions by activating the p-ERK1/2/BCL-XL pathway [175]. Metastasis-associated in colon cancer 1 (MACC1) decreases the chemosensitivity of GC to oxaliplatin by regulating FASN expression to induce anoikis resistance [176]. We concluded that FASN may be an emerging target for reversing drug resistance in cancers.

#### 4.2.3. Carnitine Palmitoyltransferase 1 (CPT1)

The maintenance of drug resistance in tumor cells is also dependent on the compensatory increase in cellular ATP and NADPH mediated by FAO [177]. CPT1 transports long-chain fatty acids from the cytoplasm to mitochondria, which is the first rate-limiting step of FAO. CPT1 is usually overexpressed in tumor cells, which exhibit increased FAO and ATP production and develop obvious resistance to glucose deprivation or hypoxic stress and chemotherapeutic drugs [178,179,180]. The CPT1 inhibitor etomoxir remarkably improved the sensitivity of drug-resistant lung adenocarcinoma cells to PTX [181].

These outcomes highlight a novel role of disrupted fatty acid metabolism in tumor lipogenesis, suggesting that key enzymes catalyzing fatty acid synthesis may be putative targets through which to overcome drug resistance in cancers.

### 4.3. Upregulation of Mitochondrial Biomass

Transcriptomic analysis revealed that the genes involved in OXPHOS and mitochondrial biotransformation were significantly upregulated in patient-derived tumor xenograft (PDX) mice with the administration of chemotherapeutic drugs. The administration of oxaliplatin and 5-FU into colon layer cultures from patients with CRC led to the enhancement of mitochondrial biomass, which was characterized by increases in mitochondrial respiratory complexes and oxygen consumption. It can be inferred that the transformation from glycolysis to OXPHOS is essential for tumor cells treated with chemotherapeutic drugs to exhibit clonal dysplasia. Moreover, the transformation from glycolysis to OXPHOS was probably mediated by the NAD^+^-dependent deacetylase sirtuin 1 (SIRT1), which increased oxidative phosphorylation by facilitating the deacetylation of peroxisome proliferator-activated receptor-gamma coactivator (PGC)-1alpha (PGC-1α). Knocking out SIRT1 and PGC-1α prevented chemotherapy-induced OXPHOS and distinctly sensitized patient-derived colonospheres as well as tumor xenografts to oxaliplatin plus 5-FU [182]. In recent years, there has been growing interest regarding mitochondrial plasticity as a key feature in cancer chemoresistance, especially mitochondrial fusion and transfer. Accordingly, mitofusin 2 (MFN2) that medicate mitochondrial fusion and OXPHOS is distinctly upregulated in surviving leukemia cells while the knockout of MFN2 rendered Jurkat sensitivity to DOX [183]. Consistently, time-dependent exposure to doxorubicin, and increased levels of MFN1, MFN2, and OXPHOS in response to doxorubicin, are usually associated with fusion-driven chemoresistance in Jurkat leukemia cells, acute myeloid leukemia (AML), and ovarian cancers [184].

Rotenone, a commonly used lipophilic pesticide, is a selective inhibitor of mitochondrial complex I. It was found that the sensitivity of HL-60 leukemia cells with rotenone-induced mitochondrial deficiency to cytarabine, ADR, PTX, and vincristine was significantly lower than that of their parental cells [185]. Dichloroacetate (DCA), a specific inhibitor of pyruvate dehydrogenase kinase (PDK), specifically acts on tumor cells with mitochondrial respiratory defects and induces citrate accumulation to activate oxidative respiration, thereby reversing 5-FU resistance in GC [186].

Mitochondrial DNA (mtDNA) encodes many proteins for the assembly and activity of mitochondrial respiratory complexes [187]. New evidence has revealed that reduced mtDNA promotes tumor survival by operating in favor of aerobic glycolysis, which is accompanied by increases in HK2 and phosphofructokinase (PFK) activities, glucose uptake, and lactic acid [188]. Reduced mtDNA also facilitated 5-FU and oxaliplatin resistance in CRC, in which aerobic glycolysis was induced and the AKT/mTOR signaling pathway was activated [189]. Consistently, mutations in mtDNA, especially trans-mitochondrial hybrids (cybrids) with mtDNA, are responsible for staurosporine, 5-FU, and CDDP resistance in pancreatic cancer [190]. Interestingly, mitochondrial transfer via exosome from fibroblasts to HeLa cells or SASr^0^ cells with depleted mtDNA restored their proliferative capacity and sensitivity to CDDP, indicating that mitochondrial transfer can be considered a potential therapeutic strategy [191]. Furthermore, mtDNA transfer in circulating TDEs from patients with hormone-therapy-resistant metastatic breast cancer induces the exit of therapy-induced cancer stem-like cells from dormancy, thus leading to endocrine resistance in OXPHOS-dependent breast cancer [192]. Although the glycolytic pathway is favored by tumor cells, the transformation from glycolysis to OXPHOS is a strong resistance-conferring event in cancers.

### 4.4. Activation of the Pentose Phosphate Pathway

Activation of the pentose phosphate pathway (PPP) is essential for pentose phosphate and ribonucleotide synthesis, which represents a driver of NADPH production. PPP activation plays a key role in tumor cells that prefer glycolysis to meet their anabolic needs and resist oxidative stress. Tumor cells exhibiting increased PPP activity were resistant to ROS-induced apoptosis, although ROS production was induced by oxidative stress, ionizing radiation, and chemotherapy [193]. PPP oxidative branches are more active in MDR cells than in drug-sensitive cells. P-gp expression and ADR resistance gradually increased, but the expression and activity of glucose-6-phosphate dehydrogenase (G6PD) and PPP metabolites decreased significantly in MCF-7 cells transformed with 3D spheroid cells. G6PD silencing increased ROS release and P-gp expression but decreased the NADPH/NADP^+^ and GSH/GSSG ratios in 2D MCF-7 cells. In contrast, the levels of ROS and P-gp decreased, but the NADPH/NADP^+^ and GSH/GSSG ratios increased in 3D MCF-7 cells overexpressing G6PD [194]. Thus, it is accepted that G6PD should be given close attention for its role in promoting drug resistance.

G6PD was identified with 3.2-fold higher level in metastatic/DOX-resistant 231-M1 than its parental 231-C3 cells, thus generating the electron-rich molecules NADPH and GSH. Enhancement in the activities of NADPH, G6PD, and 6PGD promoted DOX resistance in triple negative breast cancer [195]. Similarly, it has been demonstrated that overexpression of G6PD, 6PGD, and transketolase promotes CDDP resistance [196]. Additionally, it has been proven that G6PD and 6PGD inhibition with cytarabine exhibits synergic effects to increase anti-leukemic activities in AML [197]. DHEA, a noncompetitive inhibitor of G6PD, significantly inhibited PPP flux and NADPH production but enhanced the therapeutic potential of PTX against MDA-MB-231 cells and PDXs in the context of breast cancer [198]. Therefore, combinations of inhibitors targeting the PPP pathway with chemotherapy represent an effective strategy for cancer treatment.

Metabolic reprogramming is an important feature of drug resistance in tumors. The molecular mechanism related to drug resistance can be further clarified by discovering the dysregulated metabolic process in drug-resistant tumor cells. Targeting out-of-control metabolic processes has been shown to overcome the drug resistance of tumor cells. The metabolic enzyme-related inhibitors 2-DG, orlistat, and etomoxir have significant effects in reversing drug resistance. Phase I clinical trials for the above drugs have been carried out. It is expected that in-depth study of metabolic reprogramming in drug-resistant cells will provide a new understanding of the mechanism of drug resistance and a safer and more effective antitumor treatment strategy.

## 5. Glycosylation: A Newly Recognized Chapter in the Story of Drug Resistance

### 5.1. Glycosylation-Mediated Drug Efflux

Protein glycosylation is a well-characterized posttranslational modification, referring to the covalent attachment of single sugars or glycans to targeted proteins using glycosidic bonds. Protein glycosylation includes N-glycosylation, O-glycosylation, glycophosphatidylinositol, and c-mannosylation, among which N-glycosylation and O-glycosylation are the most well studied [199]. N-glycosylation refers to the glycosidic linkage mediated by oligo-saccharyl transferase (OST) between N-acetylglucosamine (GlcNAc) and asparagine residues through β-1,4-glycosidic bonds. O-glycosylation is distinguished by the addition of N-acetylglucosamine on serine, threonine, and tyrosine residues via UDP-GlcNAc under the action of O-GlcNAc transferase (OGT) [200]. Posttranslational glycosylation is recognized as a classic modification that occurs during drug resistance development.

Glycosylation of ABC transporters has been proven to be closely correlated with drug resistance. The overexpression of fully glycosylated MRP1 and MRP4 was associated with oxaliplatin and CDDP resistance in ovarian cancer [201]. The N-glycosylation inhibitor tunicamycin dramatically suppressed ABCG2 expression, altered its subcellular localization, and reduced the efflux of CDDP by targeting the DPAGT1/AKT/ABCG2 signaling pathway, thus reversing CDDP resistance in HCC [202]. Tunicamycin also inhibits the expression of ABCG2 and cellular translocation of P-gp in topotecan-resistant W1TR cells and PTX-resistant W1PR cells obtained from the primary ovarian cancer cell line W1 and DOX-resistant LoVo/Dx cells constructed from the colorectal cancer cell line LoVo. Tunicamycin enhances the sensitivity of these cell lines to topotecan, PTX, and DOX [203]. Swainsonine has been reported to inhibit the N-glycosylation in P-gp to downregulate its expression, thus increasing the sensitivity of Ehrlich ascites carcinoma to CDDP [204]. These findings confirmed that increased glycosylation of ABC transporters caused drug resistance, probably by increasing drug efflux, in which transcriptional regulation and posttranscriptional modifications were putative determinants. 

### 5.2. Glycosylation-Mediated Apoptosis Resistance

O-GlcNAcylation-inducing treatments with O-GlcNAcylation PUGNAc and glucosamine inhibited ERα expression, which protected MCF-7 cells from 4-OH-tamoxifen-induced apoptosis by stimulating the PI3K/AKT signaling pathway and conferred tamoxifen resistance in breast cancer [205]. OGT activity regulates miR-483-3p expression at the transcriptional level. An inhibitor of OGT activity, 2-DG, inhibits miR-483-3p expression by reducing both CTNNB1/USF1 levels and their affinity for the E-box element upstream of miR-483-3p. 2-DG induces cell apoptosis via caspase-3/7 activation, suggesting that the expression of oncogenic miR-483-3p induced by OGT activation leads to 5-FU resistance in HCC [206]. β-1,3-n-acetylglucosamine aminotransferase 8 (β3GnT8) catalyzes the biosynthesis of N-glycan branched polylactam-modified polylactosamine chains on integrin β1, thereby inhibiting oxaliplatin-mediated apoptosis and causing drug resistance in CRC [207]. More interestingly, tunicamycin not only enhances HER2^+^ breast cancer cell sensitivity to trastuzumab by inducing cell cycle arrest and apoptosis but also confers endoplasmic reticulum (ER) stress-associated apoptosis induced by ADR and vincristine in GC cells [208,209]. These results suggest that weakened apoptosis mediated by “far-reaching” glycosylation promotes drug resistance in cancers.

### 5.3. Other Mechanisms of Glycosylation Action

A growing amount of evidence has shown that other mechanisms of action of glycosylation promote drug resistance. Mucins are high-molecular-weight glycoproteins with many O-glycosylation modifications. Mucin1 (MUC1) O-glycosylation causes excessive steric hindrance, which reduces intracellular drug uptake and confers 5-FU resistance in pancreatic cancer [210]. It is well acknowledged that MUC1 overexpression mediated by O-glycosylation mitigates DNA damage and anoikis, thus leading to CDDP resistance in CRC and lung adenocarcinoma [211]. MUC1 with excessive O-glycosylation also increased the expression of MDR proteins and ultimately attenuated the sensitivity of HER2^+^ and/or ER^+^ breast cancer cells to bortezomib, trastuzumab, and tamoxifen [212]. 2-DG suppressed mucin13 (MUC13) O-glycosylation and promoted unmanaged DNA damage by inhibiting the NF-κB signaling pathway, thus enhancing the chemosensitivity of CRCs to 5-FU and CDDP [213]. β1-integrin with abundant N-glycosylation promotes EMT progression in SKBR-3 cells, therefore leading to trastuzumab and lapatinib resistance in breast cancer [214]. Guanine nucleotide-binding protein subunit beta 2-like 1 (GNB2L1) with aberrant O-glycosylation also initiates the EMT process, thereby inveigling MDR in GC [215]. DNA demethylase with abundant O-glycosylation provokes the activity of nuclear factor E2 related factor 2 (NRF2), which functions as an evolutionarily defense mechanism to counteract oxidative stress, thus conferring resistance to 5-FU in CRCs [216].

Overall, advanced glycosylation contributes to drug resistance, thus providing a theoretical basis for clinical chemosensitization. Although in vitro experiments have indicated that glycosylation inhibitors can reverse MDR, more in vivo experiments and clinical multistage trials for cancer patients are needed to confirm the effectiveness and safety of these inhibitors. The clinical application of glycosylated proteins as related targets is still challenging. Exploring the molecular mechanism underlying protein glycosylation modification and MDR can provide better strategies for developing new tumor molecular targets, evaluating tumor clinical efficacy, and reversing tumor-related MDR.

## 6. Autophagy: A Novel Force Triggering Drug Resistance

Autophagy is a biological self-digestion process devoted to degrading and recycling cellular components. Autophagy plays a role in maintaining cell stability and survival, which is largely dependent on regulation by autophagy-related proteins (ATGs). In particular, autophagy is marked by the formation of the ATG13-ULK1-FIP200 complex and is characterized by the formation of the Beclin-1-ATG14-VPS34 complex and autophagosomes related to ATG12, ATG5, and LC3II [217].

### 6.1. Protective Autophagy and Drug Resistance

Autophagy is characterized as a double-edged sword that plays a crucial role in pro-death and pro-survival processes. Recently, mitophagy has been ensured and the term mitophagy outlines the formation of autophagosome which sequesters the organelle, allowing its autophagosomal degradation following fusion with a lysosome [218]. It is well accepted that mitophagy inhibits tumorigenesis in the early stages of cancer progression, whereas it promotes tumor survival in the later stages. Importantly, it is well reported that apoptotic cell death is mediated by autophagy, which occurs prior to apoptosis in chemosensitive tumor cells but not in chemoresistant tumor cells since mitochondrial remodeling has been confirmed [219]. Typically, autophagy induced by mitochondrial remodeling often causes treatment failure that is associated with drug resistance to common chemotherapeutic drugs including CDDP, DOX, 5-FU, and PTX [220].

Tumor cells execute “protective autophagy” after being subjected to chemotherapy, thereby exhibiting distinct resistance to therapeutic drugs [221]. PTX induces upregulation of thioredoxin domain containing 17 (TXNDC17) and autophagy through Beclin-1 participation, which consequently results in PTX resistance in ovarian cancer [222]. Oxaliplatin upregulated the expression of phosphorylated ULK1, Beclin1, and ATG5 in SW480 cells. The inhibition of phosphofructokinase-2/fructose-2,6-bisphosphatase 3 (PFKFB3) reduced ULK1 phosphorylation and autophagic flux, thus enhancing the sensitivity of CRCs to oxaliplatin [223]. Gefitinib also increased the level of ROS, which in turn activated “protective autophagy”, therefore leading to mitochondrial dysfunction and gefitinib resistance in GBM and lung cancer, respectively [224,225]. Recent evidence confirmed that the heat shock protein HSP90AA1 independently acted on the PI3K/AKT/mTOR and JNK/P38 signaling pathways to trigger autophagy in osteosarcoma cells. Silencing the *HSP90AA1* gene reduces LC3II expression but increases Beclin-1 expression, thus enhancing the sensitivity of osteosarcoma cells to CDDP, DOX and methotrexate-based chemotherapy [226]. It was recently recognized that lncRNA SNHG16 upregulates ATG4B expression by sponging miR-16 to promote “protective autophagy”, ultimately touching off CDDP resistance in osteosarcoma [227]. DOX-based chemotherapy is a first-line treatment for castration-resistant metastatic prostate cancer, but its use is limited due to chemoresistance. The transcription factor forkhead box M1 (FOXM1) markedly reduced phospho-mTOR expression but enhanced the protein level of phospho-AMPK. Furthermore, FOXM1 led to DOX resistance in prostate cancer by activating the AMPK/mTOR-mediated autophagic pathway [228].

### 6.2. Protective Autophagy and Apoptosis Resistance

Autophagy can induce cancer cell death while also supporting cancer cell survival, indicating that the dual role of autophagy played in cancer therapy is associated with the regulation of apoptosis in tumor cells [229]. “Protective autophagy” has been proposed, but the mechanisms by which drug resistance is promoted have not been elucidated. However, crosstalk of other machinery between autophagy and apoptosis in chemoresistant tumor cells has been well established, whereby autophagy serves as an oncogenic factor that favors the escape of cell apoptosis [230]. It was confirmed that lncRNAs potentiated autophagy-associated drug resistance by inhibiting apoptosis. The lncRNA highly upregulated in liver cancer (HULC) was first identified to be related to the chemoresistance of HCC cells. In particular, the lncRNA HULC induces autophagy by stabilizing SIRT1. Activation of the HULC/USP22/SIRT1 signaling pathway attenuates poly (ADP-ribose) polymerase (PARP) cleavage by caspases, thus triggering 5-FU and oxaliplatin resistance in HCC [231]. Single-cell sequencing (SCS) analysis showed that lncRNA HULC and FOXM1 were aberrantly expressed in MDR GC cells and CDDP-resistant GC patients and were positively regulated by autophagic flux. These results also clarified the key role of the METase/lncRNA HULC/FOXM1 axis in reversing CDDP resistance by inhibiting autophagy but promoting apoptosis in GC [232]. The lncRNA metastasis-associated lung adenocarcinoma transcript 1 (MALAT1) acts as a competing endogenous RNA targeting miR-23b-3p and attenuates the inhibitory effect of miR-23b-3p on ATG12. The MALAT1/miR-23b-3p/ATG12 axis suppresses caspase-3/9-mediated apoptosis, thus triggering 5-FU, vincristine, and CDDP resistance in GC [233]. Epigenetic tracer analysis suggests that the activity of o-6-methylguanine-dna methyltransferase (MGMT) also potentiates autophagy by increasing ATG4B and LC3II expression, thus inhibiting PARP-mediated apoptosis and conferring CDDP resistance in GC [234]. Tripartite motif (TRIM)-containing protein 65 (TRIM65) induced the ubiquitination and degradation of TNRC6A and then suppressed the expression of miR-138-5p. Low miR-138-5p-mediated ATG7 upregulation enhanced autophagy but inhibited caspase-3-induced apoptosis, therefore facilitating CDDP resistance in NSCLC [235]. Altogether, poor responses to chemotherapy can be resolved by autophagy inhibition, which may be a novel strategy for triggering tumor apoptosis. However, further efforts should be made to elucidate the detailed mechanisms by which autophagy induces apoptosis resistance.

### 6.3. Autophagy Inhibitors in Clinical Treatment

Recently, autophagy inhibitors, such as chloroquine (CQ), hydroxychloroquine (HCQ), 3-methyladenosine (3-MA), thapsigargin (TG), and bafilomycin A1 (BAFA1), have attracted increasing attention [236]. In particular, the efficacy and adverse reactions of autophagy inhibitors have been investigated in clinical trials. A phase I/II trial in advanced BRAFV600-mutant melanoma showed that HCQ in combination with dabrafenib and trametinib every day (D+T) was well tolerated and produced an encouraging response rate (RR) and progression-free survival (PFS) [237]. Another lead-in safety study followed by a phase II trial of HCQ+D+T in patients with advanced BRAFV600-mutant melanoma also demonstrated that HCQ can prevent autophagy-driven resistance and, therefore, enhance the efficacy of BRAF-/MEK-inhibitor therapy in a “rechallenge” setting [238]. The addition of HCQ to gemcitabine or nanoparticle albumin-bound paclitaxel (nab-PTX) was also safe, which led to a greater pathological tumor response in patients with resectable pancreatic adenocarcinoma, as shown in a randomized phase II preoperative trial [239]. Additionally, the safety, pharmacokinetics, and maximum tolerated dose of CQ in combination with temozolomide have been determined in a single-center, open-label, dose-finding phase I trial for patients with newly diagnosed GBM [240]. However, potential pitfalls of these clinical trials should be discussed. First, some of these studies were nonrandomized and terminated early, partly due to poor accrual. Second, ongoing studies are currently being conducted on drug combinations for patients who are less heavily pretreated. In addition, only HCQ or chloroquine administration was examined in these trials. Whether other autophagy inhibitors can be used as attractive tools to reverse chemoresistance needs to be further studied through more clinical trials.

Currently, some classic autophagy inhibitors are in clinical trials, and new autophagy inhibitors are also actively being developed. Autophagy inhibitors have great development prospects as antitumor treatments and may become a powerful component of antitumor regimens in the future. However, contradictory research conclusions have always dominated the headlines that autophagy can either promote drug resistance or inhibit drug resistance. Furthermore, the contradiction between the drug resistance induced by autophagy and the apoptosis resistance caused by “protective autophagy” is an unavoidable problem in the context of antitumor treatment.

## 7. Conclusions and Prospects

**Figure 3:**
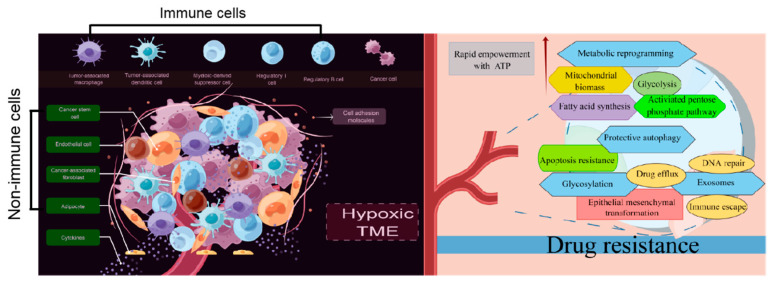
Drug resistance standing in cancers from the perspective of TME, metabolic reprogramming, exosomes, protein glycosylation, and autophagy (*By Figdraw*).

As shown in Table 1, almost all common cancers have developed drug resistance, not only to one specific drug but also to other drugs with different structures and mechanisms. Herein, we lay a foundation for a renewed understanding of drug resistance initiation and evolution from the perspective of cellular plasticity. The present data provide specific guidance on the role of the TME, metabolic reprogramming, exosomes, protein glycosylation, and autophagy in promoting drug resistance in tumor cells (Figure 3). However, other mechanisms should be followed up for a more in-depth understanding. Moreover, putative targets for clinically reversing drug resistance should be identified because all mechanisms of drug resistance development need to be considered.

## Figures and Tables

**Table 1:** A summary for anti-cancer drugs that easily develop resistance.

Anti-Cancer Drugs	Indicated Cancers
**Chemotherapeutic drugs**	
*Cisplatin*	Gastric cancer
	Breast cancer
	Head and neck squamous cell carcinoma
	Gastric cancer
	Esophageal squamous cell carcinoma
	Pleural mesotheliom
	Glioblastoma
	Osteosarcoma
	Ehrlich ascites carcinoma
	Hepatocellular carcinoma
	Ovarian cancer
*Paclitaxel*	Breast cancer
	Glioma
	Hepatocellular carcinoma
	Osteosarcoma
	Ovarian cancer
	Lung cancer
	Colorectal cancer
*Gemcitabine*	Pancreatic cancer
	Hepatocellular carcinoma
*Doxorubicin*	Gastric cancer
	Breast cancer
	Esophageal cancer
	Prostate cancer
	Bladder cancer
	Osteosarcoma
	Prostate cancer
	Multiple myeloma
	Soft tissue sarcoma
	Hepatocellular carcinoma
	Colorectal cancer
*Etoposide*	Gastric cancer
	Glioma
*Irinotecan*	Colorectal cancer
*5-fluorouracil*	Colorectal cancer
	Gastric cancer
	Pancreatic cancer
	Esophageal cancer
	Hepatocellular carcinoma
*Oxaliplatin*	Colorectal cancer
	Gastric cancer
	Prostate cancer
	Hepatocellular carcinoma
*Temozolomide*	Glioblastoma/Glioma
	Oligodendroglioma
*Vincristine*	Acute lymphoblastic leukemia
	Gastric cancer
	Oral epidermoid carcinoma
	Colorectal cancer
*Carboplatin*	Ovarian cancer
*Adriamycin*	Breast cancer
	Gastric cancer
	Osteosarcoma
	Glioma
	Burkitt lymphoma
	B-cell lymphoma
*Cytarabine*	Burkitt lymphoma
	B-cell lymphoma
*Staurosporine*	Pancreatic cancer
*Mitomycin C*	Hepatocellular carcinoma
*Camptothecin*	Hepatocellular carcinoma
*Melphalan*	Multiple myeloma
*Cyclophosphamide*	Breast cancer
*Epirubicin*	Breast cancer
**Molecular targeted inhibitors**	
*Sorafenib*	Hepatocellular carcinoma
*Tocilizumab*	Ovarian cancer
*Gefitinib*	Lung cancer
	Glioblastoma
	Breast cancer
*Trastuzumab*	Breast cancer
*Rituximab*	Chronic lymphoblastic leukemia
	B-cell lymphoma
*Cetuximab*	Oral squamous cell carcinoma
*Rapatinib*	Breast cancer
*Sunitinib*	Renal cell carcinoma
*Lapatinib*	Breast cancer
*Afatinib*	Lung cancer
*Bevacizumab*	Lung cancer
**Immune checkpoint inhibitors**	
*Nivolumab*	Lung cancer
	Melanoma
*Pembrolizumab*	Lung cancer
	Melanoma
	Colorectal cancer
	Cervical cancer
*Atezolizumab*	Lung cancer
*Ipilimumab*	Melanoma
	Lung cancer
	Gastro-esophageal cancer
*Tremelimumab*	Lung cancer
	Gastro-esophageal cancer
*Tislelizumab*	Melanoma
	Lung cancer
**Others**	
*Lenalidomide*	Multiple myeloma
*Bortezomib*	Breast cancer
	Multiple myeloma
*Tamoxifen*	Breast cancer
*Dexamethasone*	Chronic lymphocytic leukemia

## Data Availability

Not applicable.
